# Factors of COVID-19 Vaccination among Hong Kong Chinese Men Who Have Sex with Men during Months 5–8 since the Vaccine Rollout—General Factors and Factors Specific to This Population

**DOI:** 10.3390/vaccines10101763

**Published:** 2022-10-20

**Authors:** Yanqiu Yu, Rachel Hau Yin Ling, Tsun Kwan Mary Ip, Sitong Luo, Joseph T. F. Lau

**Affiliations:** 1 Department of Preventive Medicine and Health Education, School of Public Health, Fudan University, Shanghai 200032, China; 2 Key Laboratory of Public Health Safety, Fudan University, Shanghai 200032, China; 3 Centre for Health Behaviours Research, Jockey Club School of Public Health and Primary Care, The Chinese University of Hong Kong, Hong Kong SAR, China; 4 Vanke School of Public Health, Tsinghua University, Beijing 100084, China; 5 Zhejiang Provincial Clinical Research Center for Mental Disorders, The Affiliated Wenzhou Kangning Hospital, Wenzhou Medical University, Wenzhou 325000, China; 6 School of Mental Health, Wenzhou Medical University, Wenzhou 325000, China; 7 School of Public Health, Zhejiang University, Hangzhou 310058, China

**Keywords:** COVID-19 vaccination, men who have sex with men, perception, health belief model, social norm

## Abstract

This study investigated an under-researched topic regarding the prevalence of COVID-19 vaccination behavior among Chinese men who have sex with men (MSM) and the associations of this with general and MSM-specific perceptions grounded in the health belief model (HBM) and the theory of planned behaviors (TPB). A total of 400 Chinese MSM were recruited from multiple sources (site recruitment, online recruitment, and peer referral) in Hong Kong from July to October 2021, who then participated in a structured telephone interview. Of all the participants, the prevalence of COVID-19 vaccination (i.e., taking at least one dose of COVID-19 vaccination) was 78.3%. Multivariable logistic regression analyses showed that, after adjusting for background factors, (1) the general and MSM-specific HBM variables of perceived benefits and self-efficacy were positively associated with COVID-19 vaccination behavior; (2) the items or scale of general/MSM-specific perceived barriers and social norms were negatively associated with COVID-19 vaccination behavior; (3) the general perceived severity and MSM-specific perceived susceptibility, perceived severity, and cue to action were not significantly associated with COVID-19 vaccination behavior. The findings suggest that the HBM and social norm construct of the TPB only partially explained the participant’s COVID-19 vaccination behavior. Health promotion may need to focus more on modifying perceptions related to COVID-19 vaccination rather than COVID-19.

## 1. Introduction

COVID-19 vaccination is the most important measure for controlling the pandemic [1]. Relatively high vaccination rates (e.g., 80%) have been reported in some developed countries (e.g., Canada, France, Australia, and South Korea) [2]. Given the high infectivity of the new COVID-19 variants, very high uptake of COVID-19 vaccination is required to build up natural communal immunity. Disparities regarding COVID-19 vaccination in marginalized populations have raised international concerns [3,4], but this area has been under-researched.

According to the socio-ecological model, health behaviors are determined by structural factors, personal factors, and interpersonal factors [5]. Sexual minorities, such as lesbians, gay, bisexual, transgender, and queer (LGBTQ) people, are subjected to unique structural factors of health behaviors (e.g., social environment, subculture, and norm). They, in general, exhibited lower levels of health-seeking behaviors than the general population [6,7]. Their responses to the COVID-19 pandemic differed from those of the general population. For instance, the pandemic has disproportionately affected mental distress among sexual minorities due to a higher risk of unemployment [8], conflicts with their family about their sexual orientation when staying home because of social distancing [9], difficulties in finding sex partners during the pandemic [10,11], and the loss of support from sexual minority communities [9,12]. Sexual minorities were at higher risk of exposure to COVID-19 [13,14], although they tended to adhere more closely to social distancing measures than their counterparts by maintaining online social networks [15,16,17].

Despite potential disparities from mainstream heterosexual people, only two studies conducted in China have investigated COVID-19 vaccination behaviors among men who have sex with men (MSM), reporting a prevalence of 37.0% [18] and 8.7% [19], respectively. A systematic review of nine publications further reported a prevalence of vaccine acceptance of 36.0–85.4% [4]. Only two of the nine studies compared vaccine willingness between sexual minorities and heterosexual people directly and found a higher prevalence among the former than the latter [20,21]; it is plausible that sexual minorities might have more experience in adopting self-protective procedures, such as HIV testing and pre-exposure prophylaxis [22]. The review identified several factors of vaccine acceptance for COVID-19 vaccination, such as background characteristics (e.g., ethnicity) [23], medical mistrust [23], and general perceptions (e.g., perceived vaccine safety) [21,24,25]. In addition, two other studies targeting sexual minorities reported similar findings [19,24].

In the literature, the factors of COVID-19 vaccination are multi-dimensional. They commonly include perceptions related to COVID-19 (e.g., perceived risk of infection, perceived severity of COVID-19 infection, etc.) [26], perceptions related to COVID-19 vaccination (e.g., perceived effectiveness, perceived side effects, and self-efficacy) [27], trust toward the government [26], social influences (e.g., subjective norm [28]), and mental health factors (e.g., depression [29]), which are important because mental distress among MSM is disproportionately prevalent [30]. It is unclear whether these factors found in general populations would also be significant in MSM populations. Perceptions related to the disease and vaccines and perceived social support have particular importance as they have been changing during the course of the pandemic [31], and they are modifiable. This study, thus, focused on these perceptions.

There are several knowledge gaps in the literature. First, there is a dearth of studies looking at actual COVID-19 vaccination in MSM, although some studies looked at willingness regarding vaccination. Indeed, no studies have investigated COVID-19 vaccination behavior among HIV-negative MSM, while only one study has investigated COVID-19 vaccination among HIV-positive MSM, reporting a prevalence of only 8.7%; reasons against vaccination include worries about side effects and HIV disclosure [19]. Second, it is uncertain whether MSM would have high or low COVID-19 vaccination rates, as previous studies have found that MSM and the male general population differed in the levels of some COVID-19 preventive behaviors. Third, little is known about the significant factors of COVID-19 vaccination among MSM. For instance, it is unclear whether the factors of COVID-19 vaccination commonly applicable to general populations would also apply to the MSM population (e.g., perceptions related to COVID-19 and COVID-19 vaccination). Furthermore, to our knowledge, no studies have investigated the factors of vaccine acceptance specifically relevant to MSM and other sexual minorities (e.g., variables related to sexual activities). It is, thus, unknown whether the general factors and the MSM-specific factors would have independent associations with COVID-19 vaccination. Fourth, the studies related to COVID-19 vaccination among MSM were mostly conducted in Europe and the US and they tended to combine heterogeneous groups of sexual minorities, whereas MSM comprises a special population that has a disproportionately high prevalence of HIV [32] and sexual networking with multiple male sex partners [33,34], which may facilitate COVID-19 transmission. The present study filled out these knowledge gaps.

In particular, health beliefs are commonly identified determinants of COVID-19 vaccination [26,35]. The present study used the health belief model (HBM) as a conceptual framework [36]. It postulates that perceived susceptibility and perceived severity related to a health problem comprise perceived threat that would affect the health behavior of interest; in addition, perceived benefit, perceived barrier, self-efficacy, and cue to action also determine behavior. The HBM has been applied to various health behaviors related to COVID-19 (e.g., social distancing) [37,38] and is one of the most commonly applied behavioral health theories explaining influenza vaccination, HPV vaccination, and COVID-19 vaccination [26,35,39,40,41].

Health beliefs specific to MSM experiences are additional determinants of COVID-19 vaccination. MSM might perceive a higher susceptibility to COVID-19 infection than non-MSM because of their more frequent sexual networking [21,42]. MSM may also perceive specific benefits of COVID-19 vaccination, such as a reduction in risk of COVID-19 infection and anxiety during sex and facilitation in seeking a sexual partner(s). MSM might perceive more severe consequences of COVID-19 transmission as they and their male sex partners might become close contacts and require compulsory quarantine in the case of becoming infected, disclosing their same-sex behavior. Stigma during health service utilization [23] is a potential barrier against COVID-19 vaccination among MSM. Sex partners’ requests for COVID-19 vaccination is a potential cue to action. Such MSM-specific factors of COVID-19 vaccination have not been tested. The present study contended that both general and MSM-specific health beliefs would affect COVID-19 vaccination among MSM.

The HBM has been criticized for the noninclusion of interpersonal factors [43,44]. Subjective norm means a significant other’s support toward COVID-19 vaccination. It is part of the theory of planned behavior (TPB) which postulates that attitudes, subjective norms, and perceived behavioral control determine behavioral intention, which in turn determines health behavior [45]. Subjective norms were significantly associated with COVID-19 vaccination [28,46], influenza vaccination [47], and COVID-19 preventive behaviors [48,49]. Again, such potential factors have not been studied among MSM.

The present study investigated the prevalence and factors of COVID-19 vaccination among Chinese MSM in Hong Kong. The two broad types of potential factors included those that are general and MSM-specific health beliefs; both were derived from the behavioral health theories of the HBM and the TPB. Limited by the cross-sectional nature of the study, general questions on the perceived susceptibility of the HBM were not asked as vaccination would lower perceived susceptibility, which may then be a consequence instead of a factor in terms of COVID-19 vaccination.

## 2. Materials and Methods

### 2.1. Study Design and Participant Recruitment

A cross-sectional study was conducted among 400 Chinese MSM in Hong Kong. The target study population was MSM in Hong Kong. In 2015, the estimated number of MSM aged ≥20 years in Hong Kong ranged from 100,000 to 137,000 thousand [50]. Inclusion criteria were (1) Hong Kong Chinese males ≥18 years old, and (2) having had anal intercourse with at least one man in the past year.

### 2.2. Sampling and Recruitment

This study was conducted in Hong Kong from July to October 2021. Participants were recruited from multiple sources in parallel using convenient sampling. First, upon obtaining the approval of the owners, trained and experienced peer fieldworkers approached prospective MSM participants within six gay bars and four gay saunas at different time slots during weekdays and weekends; the prospective participants were briefed about the study and given an information sheet. Second, online recruitment by posting information about the study periodically, as a discussion topic on the two mainstream gay websites, was conducted; interested participants could contact the research team via private messaging or other means (e.g., phone, email, and/or social media). Third, peer referral was exercised under the guidelines of (1) previous disclosure of the eligible peer’s MSM status to the participant, (2) no previous participation in this study, and (3) an understanding of the peer’s willingness to provide the research team with his contacts. Through telephone calls, well-trained interviewers screened the eligibility of all the prospective participants. Guarantees were made on anonymity, the right to quit any time, and that refusal would not affect their chance of using any services. To maintain anonymity, only verbal informed consent was obtained; interviewers signed a form pledging that a full explanation had been given and all of their questions answered. Telephone interviews were then conducted, which took about 30 min to complete. Upon completion of the survey, an HKD 50 (USD 6.45) supermarket coupon was mailed to the participants as compensation for their time. Ethics approval was obtained from the Survey and Behavioral Research Ethics Committee of the corresponding author’s affiliated institution (SBRE-20-443).

Of the 656 MSM approached through outreaching in venues (n = 110), online recruitment (n = 525), and peer referral (n = 21), 527 were screened eligible (venue: n = 66; online: n = 455; referral: n = 16). 32 and 95 were further excluded due to not meeting inclusion criteria and refusal for participation, respectively. The final effective sample size was 400 (see Supplementary Figure S1).

### 2.3. Measures

#### 2.3.1. Background Information

This information was collected, including age, marital/cohabitation status, education level, and employment status.

#### 2.3.2. COVID-19 Vaccination Behavior

One item assessed whether the participants had taken at least one dose of COVID-19 vaccination or made an appointment in taking the first dose (yes/no response options).

#### 2.3.3. General HBM Constructs

(a) Perceived severity scale (PSEV-G): two items assessed, in the case of COVID-19 infection, the extent of negative impacts on daily life and physical health, respectively (ranging from 0 = none at all to 10 = extremely severe impacts). Cronbach’s alpha was 0.85 in this study;
(b) Perceived benefit scale (PBEN-G): one item asked “In general, COVID-19 can effectively protect myself” (ranging from 1 = strongly disagree to 5 = strongly agree);
(c) Perceived barrier scale (PBAR-G): two items assessed the level of perceived barrier: “I don’t understand enough about the side effects of COVID-19 vaccination” (PBAR1-G) and “The chance of having severe side effect after COVID-19 vaccination is higher than I could accept” (PBAR2-G) (ranging from 1 = strongly disagree to 5 = strongly agree). Due to a low level of internal consistency of these two items (i.e., Cronbach’s alpha = 0.53), no scale was formed;
(d) Self-efficacy scale (SE-G): one item was asked in this study: “If I want to take up COVID-19 vaccination, I am confident that I could do it” (ranging from 1 = strongly disagree to 5 = strongly agree).

#### 2.3.4. MSM-Specific HBM Constructs

(a) Perceived susceptibility scale specific to MSM (PSUS-MSM): one item was asked: “MSM were more likely than the general public to get infected with COVID-19” (ranging from 1 = strongly disagree to 5 = strongly agree);
(b) Perceived severity scale specific to MSM (PSEV-MSM): four items assessed various aspects of the perceived negative impacts of COVID-19 infection regarding MSM status, including (i) more severe disease outcomes than heterosexuals, (ii) a more negative experience during COVID-19 treatment than heterosexuals, (iii) worries about disclosing male sex partners as close contacts, and iv) worries about being disclosed as close contacts by infected male sex partners (ranging from 1 = strongly disagree to 5 = strongly agree). Cronbach’s alpha was 0.73 in this study;
(c) Perceived benefits scale specific to MSM (PBEN-MSM): six items assessed various potential benefits of COVID-19 vaccination that were specific to MSM, including (i) protection from COVID-19 infection during sex, (ii) effectively protecting my sex partners, (iii) being more relaxed during sex, (iv) more sexual behaviors due to my vaccination status, (v) it becoming easier to find male sex partners, and (vi) not being refused to have sexual relationships (ranging from 1 = strongly disagree to 5 = strongly agree). Cronbach’s alpha was 0.70 in this study;
(d) Perceived barriers scale specific to MSM (PBAR-MSM): two items were asked: “I am worried about being negatively treated due to my MSM status when taking up COVID-19 vaccination” and “I avoid taking up COVID-19 vaccination due to my MSM status” (ranging from 1 = strongly disagree to 5 = strongly agree). Cronbach’s alpha was 0.76 in this study;
(e) Cue to action specific to MSM (CA-MSM): one item was asked: “Some of my male sex partners require me to take up COVID-19 vaccination” (ranging from 1 = strongly disagree to 5 = strongly agree).

#### 2.3.5. General Social Norm (SN-G) and Social Norm Specific to MSM (SN-MSM)

(a) One item assessed SN-G: “Some of my good friends strongly opposed my COVID-19 vaccination” (ranging from 1 = strongly disagree to 5 = strongly agree);
(b) One item assessed SN-MSM: “My male sex partner supports me to take up COVID-19 vaccination” (ranging from 1 = strongly disagree to 5 = strongly agree).

### 2.4. Statistical Analysis

The sample size planning was conducted by using the Logistic Regression module in PASS 11.0. Assuming a prevalence of COVID-19 vaccination behavior of about 60% during the study period, the sample size of 400 would have the smallest detectable odds ratio (OR) of 1.28 (power of 0.80 and alpha of 0.05, two-sided) when these individuals were compared with those with values of the independent variable equal to the mean plus one standard deviation. The sample size is thus adequate.

Descriptive statistics are presented. A Mann–Whitney U test was conducted to test the between-group differences of the studied perceptions of COVID-19 vaccination status; effect size was estimated by *r*, with 0.1 < *r* < 0.3, 0.3 ≤ *r* < 0.5, and *r* ≥ 0.5 representing small, medium, and large effect size, respectively [51]. Univariable logistic regression analysis was conducted to test the individual associations between the general HBM constructs/social norms and COVID-19 vaccination behavior. In addition, multivariable logistic regression analysis that adjusted for the background factors was conducted to test these potential factors of COVID-19 vaccination behavior. Respectively, the crude odds ratios (ORc) and adjusted odds ratios (ORa) and their 95% confidence intervals (CI) were derived from the univariable and multivariable models. Statistical analysis was conducted by using SPSS 23.0. Statistical significance was defined as two-tailed *p* < 0.05.

## 3. Results

### 3.1. Descriptive Statistics

Of all the participants, the mean (SD; range) age was 32.8 (8.1; 18–68) years old. The majority were single (77.3%), had attained an educational level of college or above (87.8%), and were employed full-time (80.3%). The prevalence of COVID-19 vaccination behavior was 78.3%, i.e., having taken at least the first dose of the COVID-19 vaccination or made an appointment to do so (Table 1).

The median (inter-quartile range) scores of the general and MSM-specific cognitive factors (i.e., perceived susceptibility, perceived severity, perceived benefits, perceived barriers, cue to action, self-efficacy, and social norms) are presented in Table 2.

### 3.2. Factors of COVID-19 Vaccination Behavior

Univariate logistic regression analysis showed that only those with full-time employment (versus part-time employment) were more likely to take a COVID-19 vaccination, while the individual associations involving age, marital/cohabitation status, and educational level were statistically nonsignificant (Table 3).

Adjusted for the background factors, the multivariable logistic regression analysis presented in Table 4 showed that (1) the general and MSM-specific HBM variables for perceived benefits and self-efficacy were positively associated with COVID-19 vaccination behavior; (2) the items or scale of general/MSM-specific perceived barriers and social norms were negatively associated with COVID-19 vaccination behavior; (3) general perceived severity and MSM-specific perceived susceptibility, perceived severity, and cue to action were not significantly associated with COVID-19 vaccination behavior. It can be seen from the (Supplementary Table S1) that the effect size of the significant variables was moderate to large in magnitude, as the *r* ranged from 0.27 to 0.82.

## 4. Discussion

The present study reported a prevalence of COVID-19 vaccination of about 80% among MSM in Hong Kong. Those with full-time employment were more likely to have taken a COVID-19 vaccination, which is understandable as an electronic certificate of COVID-19 vaccination was required to enter many workplaces in Hong Kong (e.g., service industries and governmental departments); full-time people have a stronger drive to take up COVID-19 vaccination. The prevalence found in this study seems to be higher than that of 62.4% among male adults aged ≥ 18 years (aged 18–35: 67.1%; 36–45: 71.3%) reported over a similar study time period (July to October 2021) (unpublished data). The finding corroborates previous findings that sexual minorities showed stronger compliance to control measures against COVID-19 [15]. Although MSM were less likely than other males to use health services (than males of the general population [52]), no similar trend was found regarding COVID-19 vaccination.

It is plausible that the free COVID-19 vaccination process in Hong Kong, which involves simple and relatively convenient procedures (e.g., online appointment, convenient venues located in all districts, minimal interpersonal contacts, and no questions asked) and has no chance of exposing one’s MSM status, does not result in the discrimination commonly experienced by MSM when using other types of health service [24]. Interestingly, this study found that MSM-specific barriers, including worrying about being treated negatively due to participants’ MSM status and the avoidance of taking up COVID-19 vaccination because of having MSM status, were significantly associated with COVID-19 vaccination. It seems that, although discrimination during vaccination might be uncommon, self-stigma might have discouraged some MSM from taking up COVID-19 vaccination. Self-stigma is an internal perception that might not involve actual discrimination [53]. It is both a risk factor for mental distress, such as depression [54] and a barrier against the utilization of health services, such as HIV testing [55] and mental health services [56]. The contention that self-stigma instead of actual discrimination would defer COVID-19 vaccination needs to be tested in future studies. Health promotion campaigns for the primary series and booster dose of COVID-19 vaccination may need to remove self-stigma and ensure MSM that the vaccination process is discrimination-free.

It seems that MSM-specific perceived benefits related to sexual activities (e.g., protection against COVID-19 infection during sex, relaxation during sex, and ease of finding sexual partners) might have enhanced the motivation to take up COVID-19 vaccination among MSM. Such perceptions were quite common among the participants, which is understandable as previous studies found that 47.1% and 45.8% of the local MSM population had had multiple sex partners and nonregular male sex partners, respectively [57,58]. MSM might thus have sex with more than one person, some of whom might be strangers whose COVID-19 status was unknown.

Subjective norms are an important construct in the TPB. Significant others’ support toward COVID-19 vaccination was a significant factor of COVID-19 vaccination [28,46]. It was hence expected that support from both the participant’s male sexual partners and good friends were significantly associated with COVID-19 vaccination in the present study. COVID-19 vaccination requirement demanded by the participant’s sexual partners was, however, statistically nonsignificant. It is plausible that such requirements were uncommon, as seen from the low values in that scale.

Corroborating other studies [26,27,35], the general perceived benefits of protection and the general perceived barrier of concern about side effects and self-efficacy were significantly associated with COVID-19 vaccination behavior in the present study. The perceived effectiveness and perceived side effects of the vaccines and self-efficacy have clearly been identified as the key determinants of COVID-19 vaccination [27] and other types of vaccinations, such as seasonal influenza and HPV [59,60]. Considerations on the effectiveness and safety of a vaccine are also important among MSM and should be addressed in COVID-19 vaccination health promotions among MSM.

Most of the vaccination studies, including those on COVID-19 vaccination, reported significant associations between the perceived susceptibility/severity of the disease and vaccination behavior [26,35,39,40,41]. It is interesting, however, that all the variables on perceived susceptibility (MSM being more susceptible to COVID-19) and perceived severity (in general) specific to MSM were not significantly associated with vaccination behavior. The reasons for this require further investigations.

The present study has reported novel findings and filled out some knowledge gaps. First, we report a high prevalence of actual COVID-19 vaccination among MSM, which has scarcely been reported. According to the minority stress model, sexual minorities (e.g., MSM) are vulnerable to developing maladaptive cognitions/behaviors and mental distress when responding to stressors specific to their sexual minority status [61]; it is expected that such negative responses may reduce preventive behaviors such as COVID-19 vaccination among MSM. The findings, however, dispelled the concern about the disparity in vaccination rates between MSM and the mainstream general public. Interestingly, the perception that MSM care less about COVID-19 prevention might be seen as a ‘stigma’ per se. Direct comparisons of the preventive behaviors related to COVID-19 between MSM and non-MSM in the same sample is warranted to confirm whether and why MSM would take up more preventive behaviors than non-MSM, as suggested indirectly by this and other studies [52]. Second, we identified for the first time in the literature some MSM-specific factors of vaccination (e.g., enhanced sexual behaviors, perceived barriers, and support given by sex partners). This suggests that protection from COVID-19 infection during sex and the advantages of sexual behaviors are strong motivations for vaccination among MSM; these messages are potentially useful for health promotion. Third, it found that both general and MSM-specific factors may affect vaccination decisions among MSM but that MSM paid more attention to perceptions regarding the vaccines than those regarding the disease of COVID-19, as the former factors (and not the latter factors) were significant. Relatedly, in contrast to many vaccination studies conducted in the general population [26], this study found that the HBM was only partially supported among MSM. Such findings remind us that the HBM and possibly other health theories should be applied cautiously in specific populations, which may have their own specific sets of factors for COVID-19 vaccination.

Some practical recommendations based on the findings are also summarized here. First, the apparent nonexistence of any disparity implies that the government may not need to allocate extra budgets to promote COVID-19 vaccination among MSM. Yet, as 80% coverage is still inadequate to curb the pandemic and considering that COVID-19 vaccination effectiveness may wane, interventions targeting refusers and promoting boosters among MSM are still necessary. Second, the regular promotion of the vaccination used in general populations, such as those that modify the perceived benefits, perceived barriers, and self-efficacy of COVID-19 vaccination, should also be disseminated to MSM, both via general channels and gay-friendly channels (e.g., gay venues and gay-friendly social media and websites) and may involve peer opinion leaders who are MSM. Third, health promotion should integrate both general means and those tailored to MSM, as tailored interventions tended to be more effective than nontailored ones [62]. Such tailored messages stated that vaccination would enhance their sexual experiences but would not cause negative experiences as their MSM status would not be revealed during the vaccination process. Fourth, future research should investigate the roles of healthcare workers in promoting COVID-19 vaccination among MSM, as such studies do not exist. It is important to involve health workers because MSM perceiving health workers as trusted information providers regarding HPV vaccination would be helpful [63,64]. Health workers’ attitudes toward vaccination might affect vaccination behavior among MSM. Interventions may improve health workers’ awareness and sensitivity about potential hurdles against COVID-19 vaccination in sexual minority groups. Fifth, tailored vaccination promotion targeting MSM may involve peer educators (i.e., those who are also MSM) who may have a better understanding of the sex-related motivations and concerns involved (e.g., barriers). NGOs are certainly good settings to promote COVID-19 vaccination, as their workers have established rapport with MSM. They may understand the subculture and health needs of MSM and may be able to facilitate the modification of MSM-specific factors. Sixth, health promotions for vaccination may involve sex partners of MSM, as subjective norms were a significant factor. Such health promotion may be couple-based to initiate discussion about vaccination between the MSM couple or disseminate information to service users’ sex partners.

The present study has a number of limitations. First, the sampling was not random, as no sampling frame existed, and MSM is a hard-to-reach population. There are, however, many published studies targeting MSM. Notably, most of these studies used a similar convenient sampling method (to the one used in this study) to recruit participants from both social media and venues (e.g., gay-friendly bars and sauna places) [65]. Attention was paid to the recruitment procedures. Recruitment in the venues was performed by experienced peer fieldworkers, and efforts were made to ensure that the participants fulfilled the inclusion criteria. Popular gay-friendly social media websites were also approached for recruitment. The combination of the two recruitment strategies gave a better chance of recruiting a wider range of MSM to increase the sample’s representativeness as much as possible, although it is acknowledged that selection bias inevitably existed. Second, there might be participation bias. However, we could not make comparisons between the participants and nonparticipants, as the characteristics of nonparticipants were not investigated. Third, the items/scales for perceptions related to general/MSM-specific HBMs and social norms were self-constructed, as the related validated measurements were not available. Fourth, social desirability bias might exist as COVID-19 vaccination behavior was socially desirable. Fourth, due to the nature of a cross-sectional study, causal or temporal relationships cannot be inferred. Fifth, this study only looked at factors regarding perceptions of COVID-19 and COVID-19 vaccination based on the HBM and the TPB. It did not investigate other factors of COVID-19 vaccination, such as the mental health status of the MSM due to the length of the questionnaire; future studies are warranted to investigate such factors of vaccination among MSM. Last, it is a limitation that the scales regarding general and MSM-specific perceptions were developed for this study, as such scales were not available in the literature; the validity of the scales has not been tested in other studies. Another limitation was that test–retest reliability was not tested, although internal consistency statistics (Cronbach’s alpha) were used. A small pilot survey was performed on five MSM to assess the comprehensiveness of the items and obtain feedback which was used to finalize the items. Furthermore, some of these scales consisted of only one item and so construct validity could not be tested. Future studies should also develop better instruments, especially those specific to MSM.

To summarize, this study presents novel findings in the under-researched area of COVID-19 vaccination behavior among MSM. First, the prevalence was high, and no unfavorable disparity in prevalence was observed. Second, both general health beliefs (perceived benefit/barrier, self-efficacy, and subjective norms involving good friends in general) and MSM-specific health beliefs (perceived benefit/barrier and subjective norms involving male sex partners) were significantly associated with COVID-19 vaccination behavior. Thus, health promotion targeting MSM should involve both the general and MSM-specific perceptions, such as those found to be significant in this study. Third, unlike many other COVID-19 vaccination studies, perceived susceptibility and perceived severity (both in general and MSM-specific terms) were statistically nonsignificant. This implies that health promotion messages involving such perceptions about COVID-19 might not be impactful and should focus on perceptions related to COVID-19 vaccination. Furthermore, the HBM could only partially explain COVID-19 vaccination behavior among MSM. The novel findings of this study need to be confirmed by longitudinal studies and comparison studies across countries. The research question and findings might be applicable to other sexual minorities and vulnerable groups (e.g., sex workers) but require confirmation. The findings also remind us of the potential importance of self-stigma in determining COVID-19 vaccination. All in all, tailored health promotion regarding COVID-19 vaccination targeting MSM is warranted. Such interventions should take into consideration MSM-specific perceptions (e.g., perceived benefits and perceived barriers).

## Figures and Tables

**Table 1:** Descriptive statistics of the categorical variables (n = 400).

	n	%
**Background factors**		
Marital/cohabitation status		
Single	309	77.3
Living/married with male partners	79	19.8
Others ¶	12	3.0
Educational level		
Below college	49	12.3
College or above	351	87.8
Employment status		
Part-time	38	9.5
Full-time	321	80.3
Others §	41	10.3
**COVID-19 vaccination behavior ‡**		
No	87	21.7
Yes	313	78.3

Notes. ¶, Others refers to living or being married with a female or widowed. §, Others refers to unemployment, underemployment, retirement, or students. ‡, COVID-19 vaccination behavior was defined as having taken at least one dose of the COVID-19 vaccination or having made an appointment to take the first dose.

**Table 2:** Descriptive statistics of the key independent variables (n = 400).

	Range	Median (Interquartile Range)
**General HBM constructs**		
Perceived severity scale (PSEV-G)	0–10	6.0 (4.5–7.5)
Perceived benefits (PBEN-G)	1–5	4.0 (3.0–4.0)
Perceived barriers (PBAR-G)		
Insufficient understanding about side effects (PBAR1-G)	1–5	2.0 (1.0–3.0)
Unacceptable chance of severe side effects (PBAR2-G)	1–5	2.0 (1.0–3.0)
Self-efficacy (SE-G)	1–5	4.0 (2.0–4.0)
**General social norms (SN-G)**	1–5	3.0 (3.0–4.0)
**MSM-specific HBM constructs**		
Perceived susceptibility (PSUS-MSM)	1–5	1.0 (1.0–3.0)
Perceived severity scale (PSEV-MSM)	1–5	2.5 (2.0–3.0)
Perceived benefit scale (PBEN-MSM)	1–5	2.8 (2.3–3.3)
Perceived barriers scale (PBAR-MSM)	1–5	1.0 (1.0–2.0)
Cues to action (CA-MSM)	1–5	1.0 (1.0–2.0)
**MSM-specific social norms (SN-MSM)**	1–5	3.6 (3.0–4.0)

Note. HBM = health belief model; MSM = men who have sex with men.

**Table 3:** Associations between the background factors and COVID-19 vaccination.

Background Factors	ORc (95% CI)
Age	1.03 (0.99–1.06)
Marital/cohabitation status	
Single	Reference = 1.0
Living/married with male partners	0.30 (0.04–2.36)
Others ¶	0.42 (0.05–3.54)
Educational level	
Below college	Reference = 1.0
College or above	1.52 (0.78–2.98)
Employment status	
Part-time	Reference = 1.0
Full-time	2.32 (1.16–4.63) *
Others §	2.16 (0.79–5.92)

Note. ¶, Others refers to living or being married with a female or widowed. §, Others refers to unemployment, underemployment, retirement, or students. *, *p* < 0.05.

**Table 4:** Univariate and adjusted logistic regression analysis testing the associations between the HBM constructs/social norms and COVID-19 vaccination behavior among MSM (n = 400).

	COVID-19 Vaccination Behavior	
	ORc (95% CI)	ORa (95% CI)
**General HBM constructs**		
Perceived severity scale (PSEV-G)	0.99 (0.89–1.09)	1.00 (0.90–1.11)
Perceived benefits (PBEN-G)	2.42 (1.89–3.10) ***	2.38 (1.84–3.06) ***
Perceived barriers (PBAR-G)		
Insufficient understanding about side effects (PBAR1-G)	0.70 (0.57–0.85) ***	0.69 (0.56–0.85) ***
Unacceptable chance of severe side effects (PBAR2-G)	0.44 (0.35–0.55) ***	0.44 (0.35–0.55) ***
Self-efficacy (SE-G)	2.96 (2.25–3.90) ***	2.91 (2.20–3.85) ***
**General social norms (SN-G)**	0.76 (0.62–0.93) **	0.78 (0.63–0.97) *
**MSM-specific HBM constructs**		
Perceived susceptibility (PSUS-MSM)	1.19 (0.93–1.52)	1.19 (0.93–1.54)
Perceived severity scale (PSEV-MSM)	0.95 (0.71–1.26)	0.99 (0.74–1.32)
Perceived benefits scale (PBEN-MSM)	2.77 (1.86–4.13) ***	2.74 (1.82–4.12) ***
Perceived barriers scale (PBAR-MSM)	0.52 (0.38–0.71) ***	0.52 (0.38–0.72) ***
Cues to action (CA-MSM)	1.25 (0.95–1.63)	1.27 (0.96–1.68)
**MSM-specific social norms (SN-MSM)**	2.11 (1.69–2.63) ***	2.16 (1.71–2.73) ***

Notes. HBM = health belief model; MSM = men who have sex with men; ORc = crude odds ratio; ORa = adjusted odds ratio; CI = confidence interval. *, *p* < 0.05; **, *p* < 0.01; ***, *p* < 0.001. The adjusted models were adjusted for the background factors, including age, relationship status, educational level, and employment status.

## Data Availability

Data are available upon reasonable request.

## Supplementary Materials

The following are available online at https://www.mdpi.com/article/10.3390/vaccines10101763/s1. Figure S1. The flowchart of participant recruitment. Table S1. Descriptive statistics of key continuous variables and between-group difference by COVID-19 vaccination behavior.
