# Supplementary material for: Factors of COVID-19 Vaccination among Hong Kong Chinese Men Who Have Sex with Men during Months 5–8 since the Vaccine Rollout—General Factors and Factors Specific to This Population
Source: Vaccines (Basel). 2022 Oct 20;10(10):1763. doi: 10.3390/vaccines10101763 (PMC9609851; doi:10.3390/vaccines10101763)
Supplement: Supplementary file 1 [file vaccines-10-01763-s001.zip › vaccines-1908043-supplementary.pdf]

# Supplementary Material

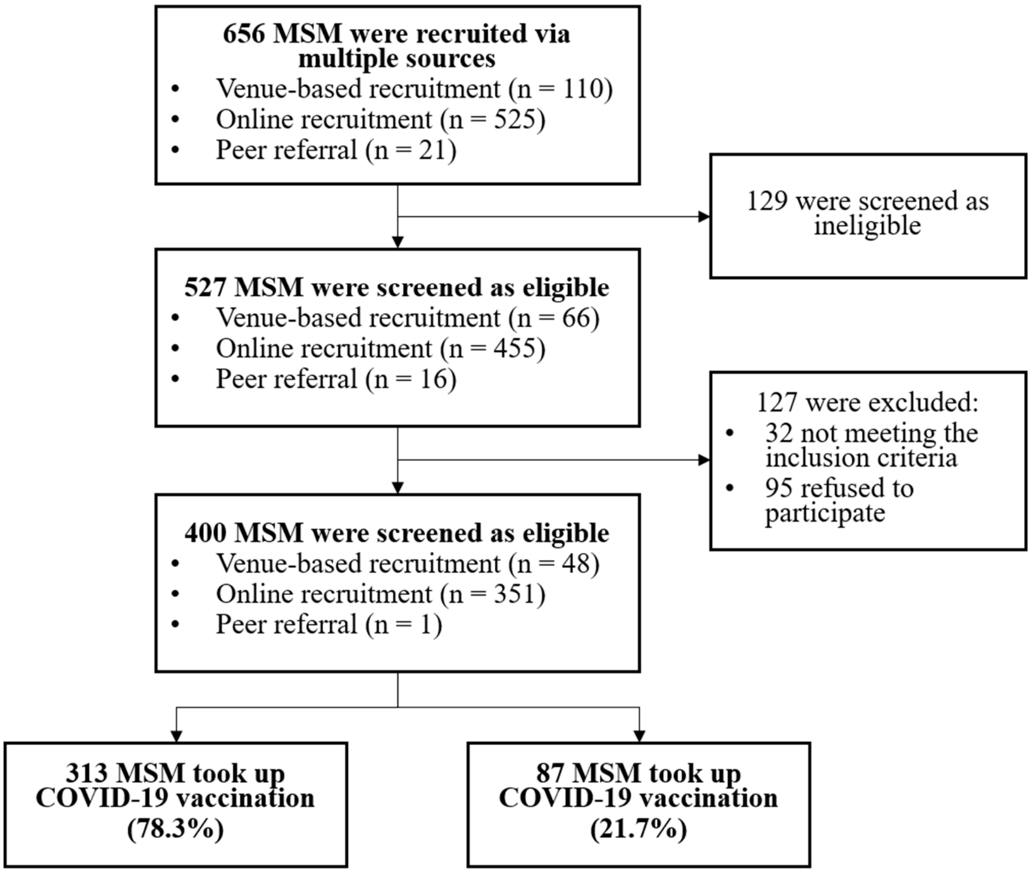


Figure S1. Flowchart of participant recruitment

Table S1 Descriptive statistics and associations between the independent variables and COVID-19 vaccination behavior

| | | Overall | COVID-19 vaccination behavior | | | | | |
|---|---|---|---|---|---|---|---|---|
| | | | Yes | | No | | | |
| | Range | Median (IQR) | Median (IQR) | Mean rank | Median (IQR) | Mean rank | *p* | *r* |
| **General HBM constructs** | | | | | | | | |
| Perceived Severity Scale (PSEV-G) | 0-10 | 6.0 (4.5-7.5) | 5.5 (4.0-7.0) | 199.3 | 6.0 (3.5-7.5) | 204.7 | 0.698 | 0.04 |
| Perceived benefits (PBEN-G) | 1-5 | 4.0 (3.0-4.0) | 3.0 (3.0-3.0) | 222.5 | 3.0 (2.0-3.0) | 121.5 | <0.001 | 0.81 |
| Perceived barriers (PBAR-G) | | | | | | | | |
| Insufficient understanding about side effects (PBAR1-G) | 1-5 | 2.0 (1.0-3.0) | 2.0 (1.0-2.0) | 190.0 | 2.0 (1.0-3.0) | 238.2 | <0.001 | 0.36 |
| Unacceptable chance of severe side effects (PBAR2-G) | 1-5 | 2.0 (1.0-3.0) | 1.0 (1.0-2.0) | 178.0 | 3.0 (2.0-3.0) | 281.6 | <0.001 | 0.82 |
| Self-efficacy (SE-G) | 1-5 | 4.0 (2.0-4.0) | 4.0 (3.0-4.0) | 222.8 | 2.0 (1.0-3.0) | 120.2 | <0.001 | 0.86 |
| **General social norms (SN-G)** | 1-5 | 3.0 (3.0-4.0) | 2.0 (1.0-3.0) | 192.7 | 3.0 (2.0-3.0) | 228.7 | 0.008 | 0.27 |
| **MSM-specific HBM constructs** | | | | | | | | |
| Perceived susceptibility (PSUS-MSM) | 1-5 | 1.0 (1.0-3.0) | 1.0 (1.0-2.0) | 205.1 | 1.0 (1.0-1.0) | 183.9 | 0.096 | 0.17 |
| Perceived Severity Scale (PSEV-MSM) | 1-5 | 2.5 (2.0-3.0) | 2.3 (1.8-2.8) | 200.1 | 2.3 (1.8-2.8) | 202.1 | 0.885 | 0.02 |
| Perceived Benefit Scale (PBEN-MSM) | 1-5 | 2.8 (2.3-3.3) | 2.8 (2.2-3.2) | 215.5 | 2.3 (1.8-2.8) | 146.5 | <0.001 | 0.51 |
| Perceived Barriers Scale (PBAR-MSM) | 1-5 | 1.0 (1.0-2.0) | 1.0 (1.0-1.5) | 189.4 | 1.0 (1.0-1.5) | 240.3 | <0.001 | 0.41 |
| Cues to action (CA-MSM) | 1-5 | 1.0 (1.0-2.0) | 1.0 (1.0-1.0) | 204.3 | 1.0 (1.0-2.0) | 187.0 | 0.163 | 0.14 |
| **MSM-specific social norms (SN-MSM)** | 1-5 | 3.6 (3.0-4.0) | 3.0 (2.0-4.0) | 221.9 | 2.0 (1.0-2.0) | 123.4 | <0.001 | 0.78 |

Note. IQR = Interquartile range. $0.1 < r < 0.3$, $0.3 \le r < 0.5$, and $r \ge 0.5$ representing small, medium, and large effect size, respectively.
